# Anxiety and Depression Prevalence and Risk Factors Among Patients With Cardiovascular Diseases in Post-COVID-19 China

**DOI:** 10.3389/fpubh.2021.758874

**Published:** 2022-01-04

**Authors:** Minglan Wu, Liying Shen, Qiqi Wang, Li Liu, Sen Lu, Jianmei Jin, Zhen Dai, Zheyue Shu

**Affiliations:** ^1^Department of Clinical Pharmacy, The First Affiliated Hospital, Zhejiang University, School of Medicine, Hangzhou, China; ^2^Zhejiang Provincial Key Laboratory for Drug Evaluation and Clinical Research, Hangzhou, China; ^3^College of Medicine, Affiliated Huzhou Hospital, Zhejiang University, Huzhou, China; ^4^Department of Cardiology and Atrial Fibrillation Center, The First Affiliated Hospital, School of Medicine, Zhejiang University, Hangzhou, China; ^5^Library, The First Affiliated Hospital, School of Medicine, Zhejiang University, Hangzhou, China; ^6^Department of Colorectal Surgery, The First Affiliated Hospital, Zhejiang University, School of Medicine, Hangzhou, China; ^7^Division of Hepatobiliary and Pancreatic Surgery, Department of Surgery, The First Affiliated Hospital, Zhejiang University, School of Medicine, Hangzhou, China

**Keywords:** cardiovascular disease, COVID-19, anxiety, depression, risk factors

## Abstract

**Objective:** Data are limited on the psychological disorders of patients with cardiovascular disease during the post-COVID-19 period, although mental health status is associated with morbidity and mortality. We aimed to investigate the prevalence of anxiety and depression and risk factors among patients with cardiovascular disease in the post-pandemic period.

**Method:** A cross-sectional survey was conducted through opportunistic and snowball sampling in southeast China from 10 October to 24 November. Anxiety and depression were assessed on the hospital anxiety and depression scale (HADS).

**Results:** A total of 435 patients with hypertension (48.05%), atrial fibrillation (17.24%), coronary artery disease (14.48%), heart failure (9.89%) and other heart diseases (10.34%) completed the survey. Interestingly, most patients reported monthly income comparable to (90.11%) or even greater than (8.51%) pre-pandemic income. The occurrence of anxiety and depression was 11.72 and 9.20%, respectively. Marital status and treatment interruption during the pandemic were independent risk factors for both anxiety and depression. Moreover, current monthly income and access to telemedicine during the pandemic were independent risk factors for anxiety.

**Conclusion:** Patients with cardiovascular disease may experience anxiety and depression not only because of disease complications but also because of the effects of the pandemic. In facing the global challenge posed by the coronavirus, efforts should be made to improve patients' psychological well-being in the management of populations with cardiovascular disease.

## Introduction

COVID-19 was declared a pandemic by the World Health Organization in March of 2020 and continues worldwide (5). First identified in Wuhan, China, in December of 2019, COVID-19 rapidly affected the entire country and then spread to the rest of the world. To effectively stem the spread of the virus, the Chinese government swiftly adopted rigorous and comprehensive measures including enforced quarantine and isolation, and mobilization of medical resources across the country (2). Since April 29th 2020, the lockdown restrictions have gradually been lifted, and virus control entered the post-pandemic stage in China (3).

Cardiovascular diseases are a leading public health problem worldwide (4). Increasing evidence indicates that patients with cardiovascular disease have a higher rate of psychological disorders than is found in the general population, and mental health status is associated with cardiovascular disease morbidity and mortality (6, 7). Previous studies have shown that large-scale disasters such as major health emergencies may trigger stress related disorders such as anxiety, sadness, insomnia and confusion (7, 8). Understanding patients' psychological distress and behavioral responses to emerging infectious diseases may be important in cardiovascular disease management (9). A variety of studies have focused on the effects of COVID-19 on the public during lockdown (10, 11); however, data on the prevalence of psychological disorders in patients with cardiovascular disease in the mitigation stage of the pandemic are limited.

In this cross-sectional study, we aimed to address the rates of anxiety and depression in patients with underlying cardiovascular disease, and to explore the related risk factors during the post-COVID-19 period in China.

## Methods

### Study Population and Inclusion Criteria

**Figure 1:**
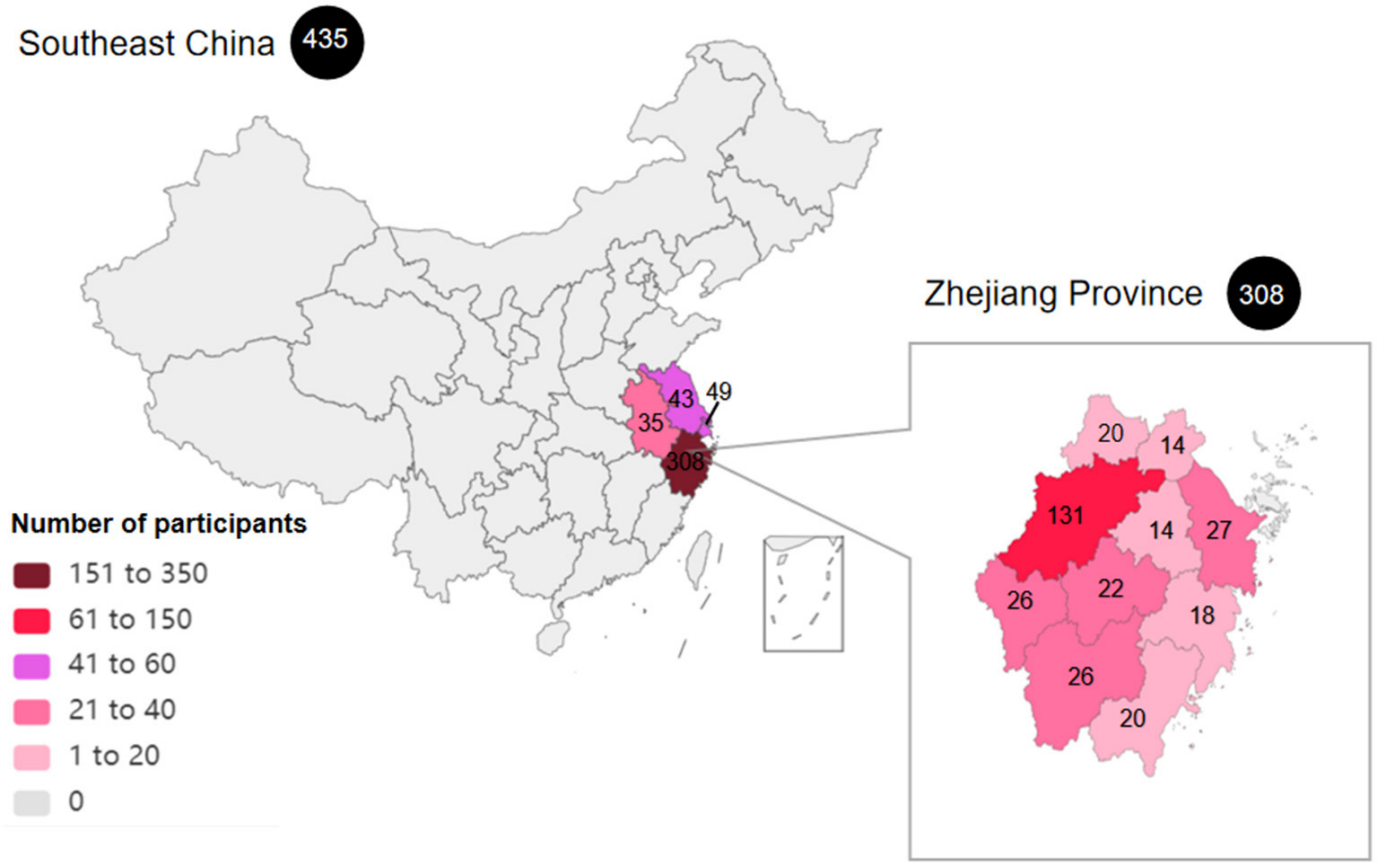
The regional distribution of the patients included in the study. A total of 435 respondents were included in this study from four provinces (Zhejiang, Anhui, Jiangsu and Shanghai) in southeast China. Most participants were from Zhejiang Province.

The participants of this cross-sectional survey were recruited from outpatients with cardiovascular disease in the Department of Cardiology of the First Affiliated Hospital, School of Medicine, Zhejiang University, a 5000-bed tertiary hospital and a major referral center between 10 October 2020 and 24 November 2020, using opportunistic and snowball sampling. The participants came from four provinces (Zhejiang, Anhui, Jiangsu and Shanghai) in southeast China, majority (70.80%) were from Zhejiang Province. The regional distribution of the respondents is presented in Figure 1. After scanning a quick response code with their mobile phones, the participants completed the questionnaire in Chinese through an online survey. Illiterate patients were asked to answer orally and have their family members fill in the questionnaire on their behalf.

The inclusion criteria were as follows: (1) patients diagnosed by their managing cardiologists as having cardiovascular disease (including hypertension, coronary artery disease, arrhythmias, heart failure and other heart diseases) according to internationally accepted criteria and (2) patients older than 18 years of age who were current residents of China. Patients were excluded if they could not comprehend the questionnaire or refused to provide consent. The research proposal was approved by the Clinical Research Ethics Committee of the First Affiliated Hospital, College of Medicine, Zhejiang University, on 9 October 2020 (approval No. 2020-EC-598). Informed consent was obtained before all patients voluntarily participated in the survey.

The survey was conducted when local work and daily life gradually returned to normal in most areas of the mainland, while the Chinese government strictly continued virus control for key regions, key target groups and inbound cases (3).

### Measures

We designed a two-part questionnaire for this study. First, social-demographic characteristics, clinical information on the participants and their medical experiences during the pandemic were assessed. The second part of the questionnaire comprised the Hospital Anxiety and Depression Scale (HADS) questionnaire.

The HADS questionnaire was used to investigate the rate of anxiety and depression in the participants. It comprises 14 questions: seven associated with the anxiety evaluation (HADS-A) and seven associated with the depression evaluation (HADS-D). Each item was rated on a 4-point scale ranging from 0 to 3. For both HADS-A and HADS-D, we divided the respondents into subgroups by using a cut-off of ≥8 points for depression or anxiety to define pathologic and non-pathologic values, according to the recommendations in the literature (12, 13). Good reliability and validity of the HADS have been reported in a prior study conducted among Chinese residents with cardiovascular diseases (14), in which Cronbach's α values of the HADS, HADS-A subscale and HADS-D subscale were 0.879, 0.806, and 0.806 respectively, and the intra-class correlation coefficients were 0.945, 0.921, and 0.932, respectively. In the current study, the reliability of the HADS was even better, with Cronbach's α coefficients of 0.889 (total scale), 0.804 (HADS-A subscale) and 0.812 (HADS-D subscale).

### Statistical Analysis

Statistical analysis was performed in SPSS Statistics v. 22.0 (International Business Machines Corp, Armonk, NY, USA). The normally continuous variables were summarized as mean ± SD (standard deviation) and compared with Student's *t*-test. For non-normally distributed data, continuous variables were summarized as medians with interquartile ranges (25th and 75th percentiles), and differences in continuous variables were analyzed with the Mann–Whitney U test. Categorical data were reported as the *n* (%) and compared with Pearson's chi-square test. Independent risk factors for anxiety and depression were assessed with multivariable logistic regression analysis. First, we used univariable analysis to screen the candidate influencing factors, and only variables with *P* <0.10 were included in multivariable logistic regression models. *P* < 0.05 was considered statistically significant.

## Results

### Baseline Demographics

A total of 474 respondents were recruited, among which 435 (91.77%) were valid for analysis. During the period of our survey, there were 53,360 outpatients in our Department of Cardiology, with a participation rate of 0.82%.

Reasons for exclusion were age younger than 18 years (*n* = 1) and the presence of other diseases without cardiovascular disease (*n* = 38). The social-demographic characteristics of respondents are presented in Table 1. In the total sample (*n* = 435), 139 (32.18%) participants were male, and 293 (67.82%) were female. The mean age of the participants was 63.25 years (SD = 9.76). The cardiovascular disease was characterized as follows: 209 (48.05%), 75 (17.24%), 63 (14.48%) and 43 (9.89%) patients had hypertension, atrial fibrillation, coronary artery disease and heart failure, respectively, and 45 (10.34%) respondents had other heart diseases including rheumatic heart disease, heart valve disease, arrhythmia or cardiomyopathy. A total of 53 (12.18%) patients had other comorbidities with cardiovascular disease, such as cancer, diabetes or other chronic diseases.

**Table 1:** Baseline characteristics of patients with cardiovascular disease.

**Characteristic**	**Patients (*n* = 435)**	**Characteristic**	**Patients (*n* = 435)**
Age (years), mean ± SD	63.25 ± 9.76	Comorbidities^a^	
Sex		Yes	53 (12.18)
Male	139 (32.18)	No	382 (87.82)
Female	293 (67.82)	Duration of disease	
Living area		<3 years	137 (33.74)
Metropolis	336 (77.60)	3–5 years	69 (17.00)
County town	29 (6.70)	5–10 years	87 (21.43)
Rural	68 (15.70)	>10 years	113 (27.83)
Educational level		Disease control	
Primary school or below	56 (12.93)	Good	335 (90.79)
Middle school	225 (51.96)	Bad	34 (9.21)
College or bachelor's degree	145 (33.49)	HADS-A score, median (IQR)	3 (1, 6)
Master's degree or above	7 (1.62)	HADS-D score, median (IQR)	2 (0,5)
Marital status		Anxiety^b^	
Single	5 (1.17)	Yes	51 (11.72)
Married	387 (90.85)	No	384 (88.28)
Divorced/widowed	34 (7.98)	Depression^c^	
Cardiovascular disease		Yes	40 (9.20)
Hypertension	209 (48.05)	No	395 (90.80)
Atrial fibrillation	75 (17.24)		
Coronary artery disease	63 (14.48)		
Heart failure	43 (9.89)		
Other heart diseases	45 (10.34)		

*Values are number (percentage) unless otherwise noted*.

*SD, standard deviation; HADS-A, hospital anxiety and depression scale-anxiety; HADS-D, hospital anxiety and depression scale-depression; IQR, interquartile range*.

a *Comorbidities such as cancer, diabetes and other chronic diseases;*

b *HADS-A score ≥8;*

c *HADS-D score ≥8*.

### The Occurrence of Anxiety and Depression in the Patients

As shown in Table 1, the median HADS-A score of participants with cardiovascular disease was 3 (1,6). According to the cut-off points, 51 (11.72%) patients had anxiety. The median HADS-D score of respondents was 2 (0, 5). According to the categorization of the HADS-D score, 40 (9.20%) patients had depression.

### Social and Clinical Risk Factors Associated With Anxiety and Depression

To identify the factor influencing the psychological status, we divided the participants into subgroups with anxiety/non-anxiety and depression/non-depression. Social and clinical risk factors associated with depression and anxiety are summarized in Table 2. Compared with the patients in the non-anxiety/non-depression group, both anxiety and depression were more common in patients who were living in county town or rural areas (39.22 and 47.50%, respectively), who were single/divorced/widowed (17.64 and 17.50%, respectively) and who had an educational level of primary school or below (25.49 and 32.50%, respectively).

**Table 2:** Comparison of social and clinical features in patients in the anxiety/non-anxiety and depression/non-depression groups.

**Characteristic**	**Anxiety**		***P-*value**	**Depression**		***P-*value**
	**No (*n* = 384)**	**Yes (*n* = 51)**		**No (*n* = 395)**	**Yes (*n* = 40)**	
Age (years), mean ± SD	63.54 ± 9.259	61.12 ± 12.804	0.197	63.47 ± 9.119	61.08 ± 14.060	0.314
Sex			0.851			0.449
Male	122 (32.02)	17 (33.33)		124 (31.63)	15 (37.50)	
Female	259 (67.98)	34 (66.67)		268 (68.37)	25 (62.50)	
Living Area			**0.003**			**<0.001**
Metropolis	305 (79.84)	31 (60.78)		315 (80.15)	21 (52.50)	
County town	21 (5.50)	8 (15.69)		24 (6.11)	5 (12.50)	
Rural	56 (14.66)	12 (23.53)		54 (13.74)	14 (35.00)	
Educational level			**0.031**			**<0.001**
Primary school or below	43 (11.26)	13 (25.49)		43 (10.94)	13 (32.50)	
Middle school	205 (53.66)	20 (39.22)		209 (53.18)	16 (40.00)	
College or bachelor's degree	128 (33.51)	17 (33.33)		136 (34.61)	9 (22.50)	
Master's degree or above	6 (1.57)	1 (1.96)		5 (1.27)	2 (5.00)	
Marital status			**0.002**			**<0.001**
Single	2 (0.53)	3 (5.88)		2 (0.52)	3 (7.50)	
Married	345 (92.00)	42 (82.35)		354 (91.71)	33 (82.50)	
Divorced/widowed	28 (7.47)	6 (11.76)		30 (7.77)	4 (10.00)	
^a^Comorbidities			0.922			0.949
Yes	47 (12.24)	6 (11.76)		48 (12.15)	5 (12.50)	
No	337 (87.76)	45 (88.24)		347 (87.85)	35 (87.50)	
Duration of disease			**0.026**			0.326
<3 years	115 (32.30)	22 (44.00)		121 (32.97)	16 (41.00)	
3–5 years	56 (15.73)	13 (26.00)		60 (16.35)	9 (23.10)	
5–10 years	82 (23.03)	5 (10.00)		82 (22.34)	5 (12.80)	
>10 years	103 (28.93)	10 (20.00)		104 (28.34)	9 (23.10)	
Disease control			**0.017**			0.121
Good	295 (92.19)	40 (81.63)		304 (91.57)	31 (83.80)	
Bad	25 (7.81)	9 (18.37)		28 (8.43)	6 (16.20)	

*Values are number (percentage) unless otherwise noted*.

*SD, standard deviation*.

a *Comorbidities such as cancer, diabetes and other chronic diseases*.

*The bold values all highlight P < 0.05, which are considered statistically significant*.

Poor disease control was noted in nine (18.37%) patients in the anxiety group and 25 (7.81%) patients in the non-anxiety group (*P* = 0.017). A significantly greater proportion of patients in the anxiety group than the non-anxiety group had a disease duration below 5 years (70%) and a monthly income of either $454 or below (48.00%) or $757 to $1,513 (32.00%). However, compared with those in the non-depression group, the patients in the depression group had a higher frequency of monthly income of $454 or below (50.00%) or $1,513 or above (7.50%).

### COVID-19-Associated Questions and Associations With Anxiety and Depression

COVID-19-associated questions in the two subgroups are presented in Table 3. A total of 392 (90.11%) and 37 (8.51%) patients reported monthly income comparable to or higher than their pre-pandemic income respectively.

**Table 3:** COVID-19-associated questions among patients with cardiovascular disease.

**Characteristic**	**Total (*n* = 435)**	**Anxiety**		***P-*value**	**Depression**		***P-*value**
		**No (*n* = 384)**	**Yes (*n* = 51)**		**No (*n* = 395)**	**Yes (*n* = 40)**	
Treatment interruption during pandemic				**<0.001**			**0.001**
Yes	50 (11.57)	33 (8.66)	17 (33.33)		39 (9.95)	11 (27.50)	
No	382 (88.43)	348 (91.34)	34 (66.67)		353 (90.05)	29 (72.50)	
Relatives or friends experienced COVID-19				0.245			0.307
Yes	3 (0.69)	2 (0.52)	1 (1.96)		3 (0.76)	0 (0)	
No	430 (99.31)	380 (99.48)	50 (98.04)		390 (99.24)	40 (100.0)	
Income fluctuation before and after COVID-19 outbreak				0.463			0.506
Stable	392 (90.11)	347 (90.36)	45 (88.24)		354 (89.62)	38 (95.00)	
Increase	37 (8.51)	6 (1.56)	0/0.0		6 (1.52)	0 (0)	
Decrease	6 (1.38)	31 (8.07)	6 (11.76)		35 (8.86)	2 (5.00)	
Current monthly income				**<0.001**			**<0.001**
< $454	81 (19.76)	57 (15.83)	24 (48.00)		61 (16.49)	20 (50.00)	
$454–$757	177 (43.17)	169 (46.94)	8 (16.00)		169 (45.68)	8 (20.00)	
$757–$1513	122 (29.76)	106 (29.44)	16 (32.00)		113 (30.54)	9 (22.50)	
>$1513	30 (7.32)	28 (7.78)	2 (4.00)		27 (7.30)	3 (7.50)	
Duration of treatment during pre-pandemic				0.179			0.21
<1 month	88 (20.32)	74 (19.37)	14 (27.45)		76 (19.34)	12 (30.00)	
1–3 months	153 (35.33)	132 (34.55)	21 (41.18)		138 (35.11)	15 (37.50)	
3 months−1 year	74 (17.09)	66 (17.28)	8 (15.69)		67 (17.05)	7 (17.50)	
Hardly ever	118 (27.25)	110 (28.80)	8 (15.69)		112 (28.50)	6 (15.00)	
Duration of treatment during pandemic				0.395			0.468
<1 month	62 (14.39)	53 (13.95)	9 (17.65)		53 (13.55)	9 (22.50)	
1–3 months	164 (38.05)	142 (37.37)	22 (43.14)		150 (38.36)	14 (35.00)	
3 months−1 year	86 (19.95)	75 (19.74)	11 (21.57)		78 (19.95)	8 (20.00)	
Hardly ever	119 (27.61)	110 (28.95)	9 (17.65)		110 (28.13)	9 (22.50)	
Access to telemedicine during pre-pandemic				0.288			**0.026**
Had used it	51 (11.92)	42 (11.14)	9 (17.65)		41 (10.57)	10 (25.00)	
Knew about but never used it	266 (62.15)	234 (62.07)	32 (62.75)		244 (62.89)	22 (55.00)	
Never heard about it	111 (25.93)	101 (26.79)	10 (19.61)		103 (26.55)	8 (20.00)	
Access to telemedicine during pandemic				**0.03**			**<0.001**
Had used it	73 (17.26)	58 (15.59)	15 (29.41)		57 (14.88)	16 (40.00)	
Knew about but never used it	266 (62.88)	236 (63.44)	30 (58.82)		246 (64.23)	20 (50.00)	
Never heard about it	84 (19.86)	78 (20.97)	6 (11.76)		80 (20.89)	4 (10.00)	

*Values are number (percentage) unless otherwise noted*.

*The bold values all highlight P < 0.05, which are considered statistically significant*.

Both anxiety and depression were more common in patients who experienced treatment interruption during the pandemic (33.33 and 27.50%, respectively) and who used telemedicine more frequently during the pandemic (29.41 and 40.00%, respectively) than in patients in the non-anxiety/non-depression group. More frequent use of telemedicine during the pre-pandemic period was observed in the depression group than the non-depression group (25.00 vs 0.10.57%, *P* = 0.026).

### Independent Risk Factors Associated With Anxiety and Depression

Multivariable logistic regression analysis identified that marital status, current monthly income, treatment interruption during the pandemic and access to telemedicine during the pandemic were independent risk factors for anxiety (Table 4). Likewise, multivariable logistic regression analysis indicated that marital status and treatment interruption during the pandemic were independent risk factors for depression (Table 5).

**Table 4:** Multivariable logistic regression analysis of factors associated with anxiety.

**Characteristic**	**OR**	**95% CI**	***P-*value**
Living area			0.06
Metropolis	1	Reference	
County town	1.613	0.555–4.690	0.380
Rural	4.513	1.278–15.933	0.019
Educational level			0.815
Primary school or below	1	Reference	
Middle school	0.424	0.022–8.293	0.572
College or bachelor's degree	0.684	0.041–11.394	0.791
Master's degree or above	0.807	0.05–13.088	0.880
Marital status			**0.008**
Single	1	Reference	
Married	4.349	0.364–51.991	0.246
Divorced/widowed	0.253	0.07–0.914	0.036
Duration of disease			0.144
<3 years	1	Reference	
3–5 years	1.242	0.458–3.37	0.67
5–10 years	1.974	0.672–5.797	0.216
>10 years	0.385	0.099–1.490	0.167
Disease control	0.892	0.272–2.921	0.85
Current monthly income			**0.022**
< $454	1	Reference	
$454–$757	7.496	0.077–732.635	0.389
$757–$1513	0.259	0.003–19.337	0.539
>$1513	5.430	0.175–168.413	0.334
Treatment interruption during pandemic	4.042	1.529–10.686	**0.005**
Access to telemedicine during pandemic			**0.031**
Had used it	1	Reference	
Knew about but never used it	1.24	0.499–3.086	0.643
Never heard about it	0.228	0.06–0.858	0.029

*OR, odds ratio; CI, confidence Interval*.

*The bold values all highlight P < 0.05, which are considered statistically significant*.

**Table 5:** Multivariable logistic regression analysis of factors associated with depression.

**Characteristic**	**OR**	**95% CI**	***P-*value**
Living area			0.585
Metropolis	1	Reference	
County town	0.750	0.269–2.091	0.583
Rural	1.457	0.404–5.259	0.566
Educational level			0.362
Primary school or below	1	Reference	
Middle school	0.311	0.027–3.626	0.351
College or bachelor's degree	0.260	0.028–2.376	0.233
Master's degree or above	0.158	0.019–1.341	0.091
Marital status			**0.008**
Single	1	Reference	
Married	6.266	0.580–67.651	0.131
Divorced/widowed	0.326	0.091–1.166	0.085
Current monthly income			0.353
< $454	1	Reference	
$454–$757	0.103	0.001–20.716	0.401
$757–$1,513	0.022	0.00–3.666	0.144
>$1,513	0.201	0.003–13.456	0.454
Treatment interruption during pandemic	2.924	1.025–8.342	**0.045**
Access to telemedicine during pre-pandemic			0.988
Had used it	1	Reference	
Knew about but never used it	0.899	0.224–3.602	0.88
Never heard about it	0.884	0.146–5.351	0.893
Access to telemedicine during pandemic			0.203
Had used it	1	Reference	
Knew about but never used it	0.629	0.168–2.362	0.492
Never heard about it	0.174	0.024–1.241	0.081

*OR, odds ratio; CI, confidence Interval*.

## Discussion

During the COVID-19 pandemic, most people experience psychological pressure because of the uncertainty of the development of the pandemic, fear of infection and decreased social activities (15). Little is known about the presence of anxiety and depression among individuals with cardiovascular disease, particularly in the post-pandemic stage.

The results of this current study indicated 11.72 and 9.20% occurrence of anxiety and depression in participants, respectively. Compared with data from the peak stage of the COVID-19 outbreak reported in other studies (18.5–23.5% occurrence of anxiety/depression), the prevalence of psychological disorders among patients with cardiovascular disease appeared to slightly decrease post-pandemic (16, 17). These results are consistent with findings from a study by Chen et al. (18) in Hubei, China, reporting that the prevalence of psychological distress decreased from more than 75% to approximately 15% during the peak and mitigation stages of the pandemic. Because psychological disorders are highly associated with cardiovascular disease morbidity and mortality (5, 13), the importance of patient mental health during the post-pandemic stage should be emphasized. Previous studies have focused only on the effects of temporal lockdown on patients with cardiovascular disease. Here, we provide initial information on patients' mental well-being during the long post-pandemic stage.

To better manage psychological disorders in patients with cardiovascular disease, emerging studies have reported on risk factors for anxiety and depression, and have indicated that female sex (19), low education (6), persistent complications and weakened physical function (20) are risk factors. The present study revealed that marital status was an independent risk factor for both anxiety and depression, and current monthly income was a risk factor for anxiety among patients with cardiovascular disease. Interestingly, most of the respondents had comparable or even greater income than pre-pandemic levels. The Chinese authorities' decisive actions and targeted measures against COVID-19, and the restoration of industrial chains, somewhat mitigated the effects of the crisis.

We observed no significant differences between the anxiety and non-anxiety cardiovascular disease subgroups in having relatives or friends who contracted COVID-19. However, the number of participants was limited, so these results may not robustly reflect real-world conditions.

We found that both anxiety and depression were more frequent in patients experiencing treatment interruption during the pandemic. Thus, ensuring a timely medical service is essential for individuals with cardiovascular disease during the pandemic. Access to telemedicine during the pandemic was confirmed to be an independent risk factor for anxiety in our research. The results of this study also suggest that the COVID-19 crisis has accelerated the spread of telemedicine. More participants have used or heard about telemedicine after the outbreak of the COVID-19 pandemic. Telemedicine may compensate for traditional health care in the management of patients with cardiovascular disease during the pandemic.

## Limitations

Although this study is the first analysis addressing the prevalence of anxiety and depression in patients with cardiovascular disease during the mitigation stage of the COVID-19 pandemic, it has some limitations. First, the use of online self-reported questionnaire to measure the mental health of the participants and the small participation rate may have yielded results that do not fully reflect the real-world situation. Second, the cross-sectional research design of the study does not allow for exploration of the longitudinal effects of COVID-19 on patients with cardiovascular disease. Third, the patients' current treatment regimens were not investigated in the study. Finally, this study was conducted in patients with cardiovascular disease in China, and therefore the findings may not be applicable to other populations. Because of the dearth of data in this field, this study, regardless of its limitations, should serve as an impetus for further research in other countries and other patient groups to investigate the long-term effects of the COVID-19 pandemic.

## Conclusion

Patients with cardiovascular disease may experience psychological disorders such as anxiety and depression during the post-COVID-19 period. Marital status, current monthly income, treatment interruption during the pandemic and access to telemedicine are independent risk factors. The findings of our study should be helpful in developing policies and strategies for the management of cardiovascular disease.

## Data Availability Statement

The raw data supporting the conclusions of this article will be made available by the authors, without undue reservation.

## Ethics Statement

The studies involving human participants were reviewed and approved by Clinical Research Ethics Committee of the First Affiliated Hospital, College of Medicine, Zhejiang University. The patients/participants provided their written informed consent to participate in this study.

## Author Contributions

MW and QW were involved in the conception and study design. They were responsible for data collection together with SL, JJ, ZD, and ZS. LS was involved in the writing and revision of the manuscript. LL is a biostatistician who was responsible for the data analysis. All authors were responsible for critical revision of the manuscript.

## Funding

This study was supported by the Clinical Research Fund of Zhejiang Provincial Medical Association (2018ZYC-A11), Department of Science and Technology of Zhejiang Province (grant no. LGF19H020011), and the Joint Fund of Zhejiang Provincial Natural Science Foundation (grant no. LYY19H310012), People's Republic of China.

## Conflict of Interest

The authors declare that the research was conducted in the absence of any commercial or financial relationships that could be construed as a potential conflict of interest.

## Publisher's Note

All claims expressed in this article are solely those of the authors and do not necessarily represent those of their affiliated organizations, or those of the publisher, the editors and the reviewers. Any product that may be evaluated in this article, or claim that may be made by its manufacturer, is not guaranteed or endorsed by the publisher.
